# Medium-Throughput Processing of Whole Mount *In Situ* Hybridisation Experiments into Gene Expression Domains

**DOI:** 10.1371/journal.pone.0046658

**Published:** 2012-09-28

**Authors:** Anton Crombach, Damjan Cicin-Sain, Karl R. Wotton, Johannes Jaeger

**Affiliations:** EMBL/CRG Research Unit in Systems Biology, Centre de Regulació Genòmica (CRG), and Universitat Pompeu Fabra (UPF), Barcelona, Spain; Oxford Brookes University, United Kingdom

## Abstract

Understanding the function and evolution of developmental regulatory networks requires the characterisation and quantification of spatio-temporal gene expression patterns across a range of systems and species. However, most high-throughput methods to measure the dynamics of gene expression do not preserve the detailed spatial information needed in this context. For this reason, quantification methods based on image bioinformatics have become increasingly important over the past few years. Most available approaches in this field either focus on the detailed and accurate quantification of a small set of gene expression patterns, or attempt high-throughput analysis of spatial expression through binary pattern extraction and large-scale analysis of the resulting datasets. Here we present a robust, “medium-throughput” pipeline to process *in situ* hybridisation patterns from embryos of different species of flies. It bridges the gap between high-resolution, and high-throughput image processing methods, enabling us to quantify graded expression patterns along the antero-posterior axis of the embryo in an efficient and straightforward manner. Our method is based on a robust enzymatic (colorimetric) *in situ* hybridisation protocol and rapid data acquisition through wide-field microscopy. Data processing consists of image segmentation, profile extraction, and determination of expression domain boundary positions using a spline approximation. It results in sets of measured boundaries sorted by gene and developmental time point, which are analysed in terms of expression variability or spatio-temporal dynamics. Our method yields integrated time series of spatial gene expression, which can be used to reverse-engineer developmental gene regulatory networks across species. It is easily adaptable to other processes and species, enabling the *in silico* reconstitution of gene regulatory networks in a wide range of developmental contexts.

## Introduction

One of the central challenges in biology today is to understand the structure, function, and evolution of gene regulatory networks involved in pattern formation during development. In order to achieve this, we need to map and compare spatio-temporal patterns of gene expression across different species. With the advent of high-throughput methodology, the scale at which we can generate expression data has increased dramatically. RNA-seq, DNA microarrays, and quantitative PCR are among the best known methods used for this purpose. However, none of these ‘omics’ approaches provides detailed spatial information, which is crucial in this context. Therefore, quantitative techniques based on *in situ* hybridisation (or antibody staining) combined with microscopy and image-processing algorithms have become increasingly important over the past few years [1]–[5].

We use whole mount *in situ* hybridisation (WMISH) to quantify the expression patterns of segmentation genes in early embryos of different fly species (Diptera). These genes form a regulatory network, which determines the basic body plan of the animal by creating a segmental pre-pattern of periodic gene expression along the anterio-posterior (A–P) embryonic axis [6]–[9]. Maternal mRNAs are deposited at the anterior and posterior pole of the embryo, from where their protein products diffuse to form long-range concentration gradients. These gradients are then interpreted, in a hierarchical and dynamic fashion, by the zygotic gap, pair-rule, and segment-polarity genes. We study the function and evolution of the first tier of the segmentation hierarchy—the gap gene network [10]. To achieve this, we use a reverse-engineering approach where mathematical models are fit to gene expression data to reconstitute and compare regulatory network structure and dynamics across species [11], [12].

Various methods have been developed to process and analyse images that result from ISH (or antibody staining) experiments in embryos of the vinegar fly, *Drosophila melanogaster*. They range from the analysis of high-resolution data based on fluorescent staining protocols for a relatively small set of genes [3], [13]–[22], to the high-throughput processing and analysis of ten-thousands of images for hundreds to thousands of genes [23]–[30].

In this paper, we present our own image processing and quantification pipeline, which may be characterised as a “medium-throughput” technique. It is designed for quantification of spatial expression data from a small set of genes, yet for multiple species. Compared to the high-resolution methods mentioned above, it increases robustness and versatility of the experimental protocol by using enzymatic (colorimetric) instead of fluorescent techniques (the latter being difficult to apply in non-model organisms). The speed of image acquisition and data quantification is also increased by using wide-field (rather than confocal scanning) microscopy combined with a simplified and efficient processing pipeline. Compared to the high-throughput methods mentioned above, our method allows for the measurement of graded spatio-temporal expression profiles along the A–P axis, rather than ‘on/off’ characterization and classification of 2D expression patterns on the surface of the embryo. Measurement of graded expression levels is crucial for our attempts at reverse-engineering pattern forming networks.

**Figure 1:**
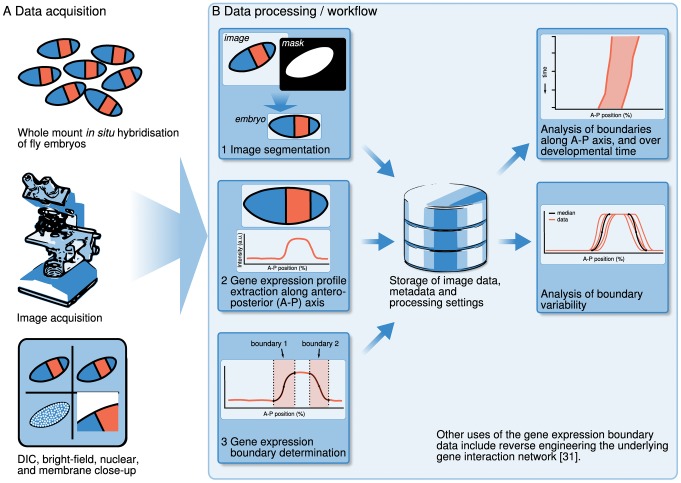
Overview of data acquisition and the data processing workflow. (A) Data acquisition consists of the following steps. First, gene expression patterns of interest are visualised using enzymatic (colorimetric) whole-mount in situ hybridisation (WMISH). Second, embryos are imaged using a compound wide-field microscope, and third, this results in a set of four images that serve as the basis for further processing. (B) Data processing consists of (1) distinguishing the embryo from the background (image segmentation), (2) extracting gene expression profiles along the A–P axis, and (3) determining the boundaries of gene expression domains using spline approximations. The resulting data, metadata, and processing parameters are stored in a database. Median boundary positions define an integrated data set of gene expression. Measured boundary profiles and positions are used to analyse variability and spatio-temporal dynamics of gene expression.

A schematic overview of our processing workflow is shown in Fig. 1. In brief, data are obtained by staining embryos for one (or two) genes of interest using WMISH. Embryos are then imaged using wide-field microscopy (Fig. 1A). Images are processed by first separating embryos from background (image segmentation), and then extracting expression profiles within a strip along the A–P axis. Finally, we identify and measure the position of individual gene expression domain boundaries by using spline approximations (Fig. 1B). Intermediate images (with associated metadata) and processing parameters are stored in a database. This results in sets of measured boundary positions from a large number of embryo images, sorted by gene and developmental time points. From these measurements, an integrated data set can be obtained by evaluating median boundary positions across space and time (Fig. 1B).

We have successfully applied our method to create spatio-temporal expression data sets for maternal and gap gene mRNA during the blastoderm stage of development in *Drosophila* and three other species of flies: the scuttle fly *Megaselia abdita*, the moth midge *Clogmia albipunctata*, and the hover fly *Episyrphus balteatus*. Currently, we are analysing the position of gene expression boundaries (Fig. 1B), and are using these data to reverse-engineer and compare the gap gene network across these species. This approach has been shown to work in *Drosophila* [31]. Equivalent analyses of the other species will be published elsewhere.

## Materials and Methods

### *in situ* Hybridisation and Microscopy

**Figure 2:**
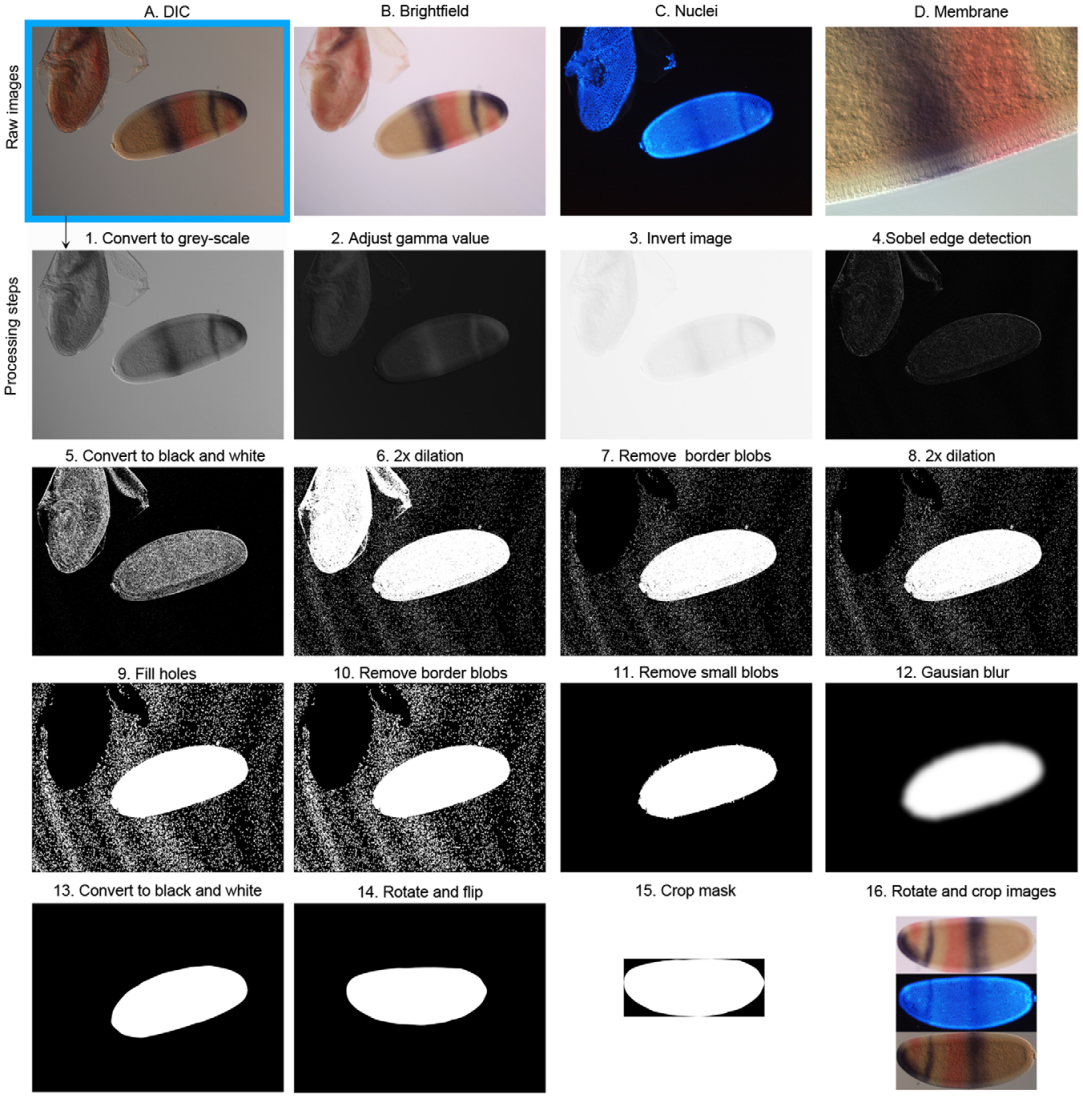
Generating the embryo mask. The top row displays the four raw images obtained by microscopy: (A) DIC, (B) bright-field, (C) nuclear counterstain, and (D) detailed membrane morphology. The DIC image is used to create the binary embryo mask. This is achieved through a series of processing steps (1–16), which are described in detail in the Materials and Methods section of the main text.

Gene expression data are acquired by means of whole-embryo enzymatic (colorimetric) *in situ* hybridisation of mRNA expression patterns. We use standard staining protocols with a few species-specific modifications, as published in [32] for *Clogmia*, [33] for *Episyrphus*, and [31] for *Drosophila* and *Megaselia*. Protocols for *Drosophila* and *Megaselia* were optimised to increase throughput without decreasing quality. All embryos were counterstained using DAPI to visualise nuclei. Raw data consists of four images acquired using a compound, wide-field fluorescence microscope: (A) a differential interference contrast (DIC) image (Fig. 2A), (B) a bright-field image (Fig. 2B), (C) a fluorescent image of the DAPI nuclear counterstain (Fig. 2C), and (D) a DIC image showing details of membrane morphology on the dorsal side of the embryo (Fig. 2D). Images A–C were acquired using a 10x objective, image D using a 40x objective. Images B and C are focused on the surface, images A and D on the sagittal plane of the embryo. All images are acquired in RGB colour mode, with each of the channels (red, green and blue) ranging in values from 0 to 255.

### Image Segmentation

Processing steps for image segmentation are shown in Fig. 2. Before we provide a step-by-step explanation of our image segmentation algorithm, we first list the general requirements for our approach. First of all, if one adheres to the following three conditions, the algorithm is guaranteed to create a good embryo mask: (1) the image contains only one embryo in its entirety, and this embryo does not touch any others, (2) all other embryos visible in the image touch the image border, and (3) the background lighting is relatively even. We stress that the algorithm is developed for DIC images. Using other sources, such as bright-field images, will work in most cases, though it is often necessary to pre-process the source images by applying an appropriate Gamma correction or other methods to increase contrast and/or luminance.

The following sequence of image segmentation operations are applied to the 10x DIC images (image A) in order to identify the embryo outline, and to separate the embryo from the background [34] (see [35] for a general description on ImageJ, the image processing framework we use; the website http://rsbweb.nih.gov/ij has detailed descriptions of ImageJ methods, macros, and plug-ins). The outcome of each step in the process is shown in Fig. 2, panels 1 to 16, and explained in the corresponding points 1 to 16 below.

1. Combine the RGB channels into a single channel by means of the ImageJ ImageConverter.convertToGray8() method.
2. Apply a gamma correction (default value 0.08) with the ImageJ ImageProcessor.gamma() method. The gamma value can be adjusted manually (see Results).
3. Invert the gray scale image to get a dark background and light foreground with the ImageJ ImageProcessor.invert() method.
4. Find edges by applying the ImageJ ImageProcessor.findEdges() method. It uses a Sobel edge detector [36] to highlight sharp changes in intensity. The brightness has been increased in panel 4 for illustrative purposes.
5. Convert to black-and-white by using the ImageJ ImageProcessor.threshold() method with the threshold parameter set to 6. All the pixels with values lower or equal to 6 are set to 0, while all the rest are set to 255. Pixels with a value of 255 are considered to be part of a ‘blob’.
6. Perform two dilations [34] using the ImageJ ImageProcessor.dilate() method.
7. Remove blobs that are touching the image border, using the ImageJ ImageProcessor.killBorderBlobs() method.
8. Perform two additional dilations using the ImageJ ImageProcessor.dilate() method.
9. Fill holes: any black areas (pixel value 0) enclosed by white pixels (255) are set to white.
10. Remove blobs that are touching the image border, as the dilations in step 8 could have generated new ones.
11. If more than one blob is present, remove the supernumerary blobs that have an area smaller than a certain threshold value. The threshold is determined by dividing the total image area by Beta, where Beta is set to an empirically determined default value of 13.0. This value can be modified in the user interface. 1.0/Beta gives the maximum blob size that should be considered non-embryo as a fraction of the image area. The result of this processing step should be an image with a single blob that segments the embryo from the background.
12. To smoothen the edges of the mask, we apply a Gaussian filter with an accuracy of 1e-3 and a standard deviation of 31 pixels along the x- and y-axis. The ImageJ ‘*Gaussian Blur*’ plug-in is used.
13. Convert the image back to black-and-white using the ImageJ ImageProcessor.threshold() method, with the threshold parameter set to 145. If more than one blob is present after thresholding, remove supernumerary blogs as indicated in step 11. This repeated blob removal operation is required because small artifactual blobs occasionally appear after blurring or thresholding.

The result of these processing steps is an image that isolates the embryo from the background: the embryo mask (Fig. 2.13). From this mask we calculate the principal axes of the embryo using statistical moments, which allows us to rotate the DIC (image A), bright-field (B), and nuclear images (C) such that the major, or antero-posterior (A–P) axis is horizontal [16].

1. Rotate the embryo mask to have the A–P axis placed horizontally. We have used an ImageJ plug-in called ‘*Orientation*’ to calculate the rotation angle with reference to the horizontal axis (in degrees). We then rotate the image by that angle in opposite direction, using the ImageJ ‘*Rotate*’ plug-in. Afterwards, the embryo may be flipped manually in the horizontal and/or vertical direction such that in the resulting dataset all embryos have the anterior facing left, and dorsal facing up.
2. Automatically crop the image to the size of the embryo mask by finding the rotated embryo mask and applying the ImageJ ImageProcessor.crop() method.
3. Rotate and crop DIC (image A), bright-field (B), and nuclear images (C) accordingly.

### Extraction of Gene Expression Profiles

**Figure 3:**
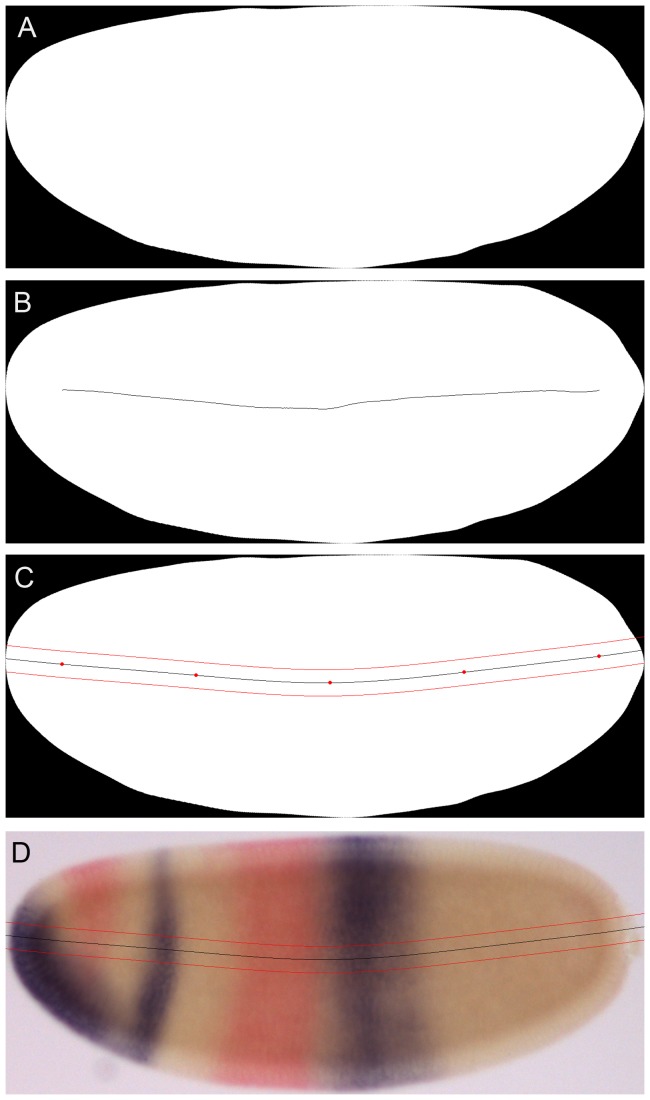
Determination of the 10% strip for profile extraction along the A–P axis. The embryo mask (A) is used to calculate the morphological skeleton shown in (B). Along this skeleton we position 5 equidistant points (red dots in C), though which we draw a cubic spline (solid black line in C). This spline is extended to the embryo borders using Lagrange extrapolation. It is then used to determine a band (or strip) that extends 10% along the minor (or dorso-ventral, D–V) axis of the embryo (5% above and below the spline; red lines in C). Expression profiles are extracted from the bright-field image by measuring the average staining intensity of vertical pixel columns that fall within the strip (D).

We calculate the skeleton of the embryo mask [34], [37], and position five equidistant points on its main branch, see figure 3. Through these points (called knots), we draw a cubic spline [37], which is extended to the embryo borders using Lagrange extrapolation, thus covering the entire A–P axis. Occasionally, minor manual adjustments are necessary to create a spline curve that is as straight and smooth as possible, yet follows the shape of the embryo. Along this smooth curve, we determine a band that extends 10% along the minor (or dorso-ventral, D–V) axis of the embryo (5% above and below the spline). From this band, we extract the RGB channels of the bright-field image by taking the average intensity per channel along the vertical columns. The signal intensity of enzymatic stains is approximated from the RGB channels: NBT/BCIP (purple stain) is extracted from the red channel (nbt_signal = red), and FastRed (red stain) is extracted by subtracting the red channel from the green (fastred_signal = green - red) [31].

### Identification of Gene Expression Boundaries

We manually determine the spatial boundaries of gene expression domains in the extracted profiles by approximating them with cubic splines that have their endknots clamped to a zero first derivative [37]. The user indicates the starting and ending point of a boundary by defining the start and end knot of the cubic spline: (x_0_,y_0_) and (x_2_,y_2_) respectively. These knots correspond to the outer and inner edges of a boundary: x_0_ marks the point at which signal can be distinguished from background noise, and x_2_ marks a position at which a high level of expression is attained, representative of expression levels in the interior of a domain. Automatically, a knot is added at (x_1_,y_1_), with x_1_ = |x_2_−x_0_|/2 and y_1_ equal to the gene expression intensity at position x_1_. As mentioned above, the spline is constrained by requiring the first derivatives at both knots to be zero (hence ‘clamped’ cubic spline). Per gene, each boundary is labelled with an integer identifier number, and whether it represents the anterior or posterior boundary of an expression domain. This enables us to compare homologous boundaries between different embryos and species, to group them according to the developmental age of an embryo, and track to expression domains over time (see Results).

### Time Classification

**Figure 4:**
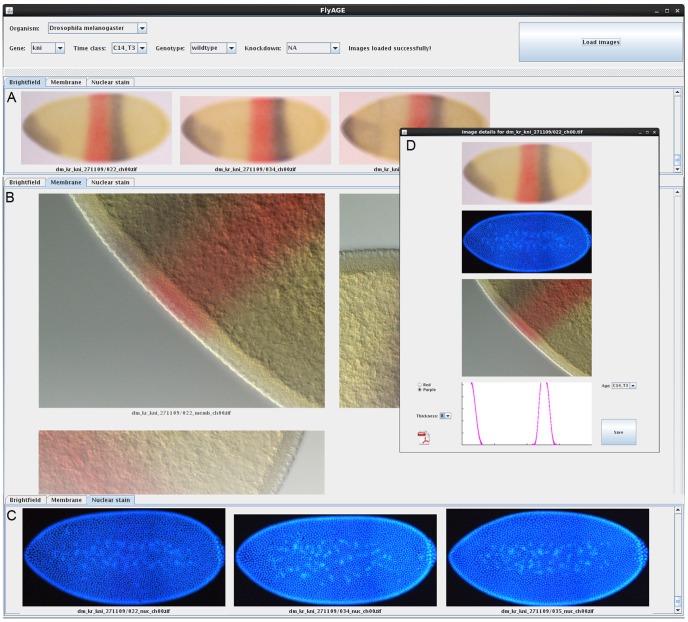
Time classification using FlyAGE. Screenshot displaying the FlyAge programme, which enables the visualisation of all embryos stained for a particular gene at a particular time class. (A) Drop-down menus at the top of the screen allow the user to select ‘Organism’, ‘Gene’, ‘Time class’, ‘Genotype’ and ‘Knock-down’. Pressing the ‘Load Images’ button displays montages of bright-field images (A), membrane details (B), and nuclear counterstains (C) in separate tabs. (D) Bright-field images, nuclear counterstain, membrane details, and expression slopes (boundaries) can be viewed together in a pop-out window for each individual embryo by clicking on the bright field image. Identified outliers, or misclassified embryos can be reclassified in this window by selecting the correct age from the ‘Time class’ drop-down menu.

Stages of development before gastrulation are divided into cleavage cycles, where cleavage cycle n is the period between mitoses n-1 and n. In the case of *Drosophila* these are named C1 to C14A. Staging by cleavage cycle is achieved by counting the number of nuclei present in the fluorescent image of the DAPI nuclear counter-stain (Figure 2C). Embryos in C14A are further subdivided into 8 time points of 6–7 min duration based on membrane morphology in the DIC image of the dorsal side of the embryo (Figure 2D). Staging follows [3] for *Drosophila*, while we use similar staging schemes for the other species (K. Wotton, A. Crombach, J. Jaeger, unpublished data). Time classification is carried out manually, after the creation of the embryo mask (see also Results). Staged embryos are inspected visually using the FlyAGE program (Fig. 4). This is done by at least 2 independent experts to avoid biases in classification.

### Analysis of Expression Boundaries

In order to visualise the expression data, we developed two tools for plotting expression domains over space and/or time. In both cases, we may select one or more gene stains (e.g. *bicoid* (*bcd*), *even-skipped* (*eve*), *hunchback (hb), knirps* (*kni*), *nubbin* (*nub*), *orthodenticle* (*otd*)), or enzymatic protein stains (shown in capitals e.g. Hunchback (HB), Caudal (Cad)) to plot. Variability plots show boundaries extracted from individual embryos, and the corresponding median boundary per time class. The media boundary is calculated by taking the median starting and end points from the data set. These plots give insight into embryo-to-embryo variability by visualizing the distribution of boundary positions along the A–P axis. They show A–P position (in percent, where 0% is anterior) on the x-axis, and normalised signal intensity on the y-axis. Space-time plots display the mid-points (x_1_) of the median slopes over time, with the A–P position on the x-, and developmental time on the y-axis (flowing downwards). Such plots allow us to observe and assess trends in the data over time.

### Implementation

We designed and implemented our workflow as a graphical user interface, named FlyGUI, in Java (http://www.java.com), using classes from the ImageJ packages (http://rsbweb.nih.gov/ij). It uses a MySQL database (http://www.mysql.com) for storage and retrieval of the data and processing settings. Source code and precompiled executables (jar files) for the Linux operating system have been made available on https://subversion.assembla.com/svn/flygui/.

## Results and Discussion

**Figure 5:**
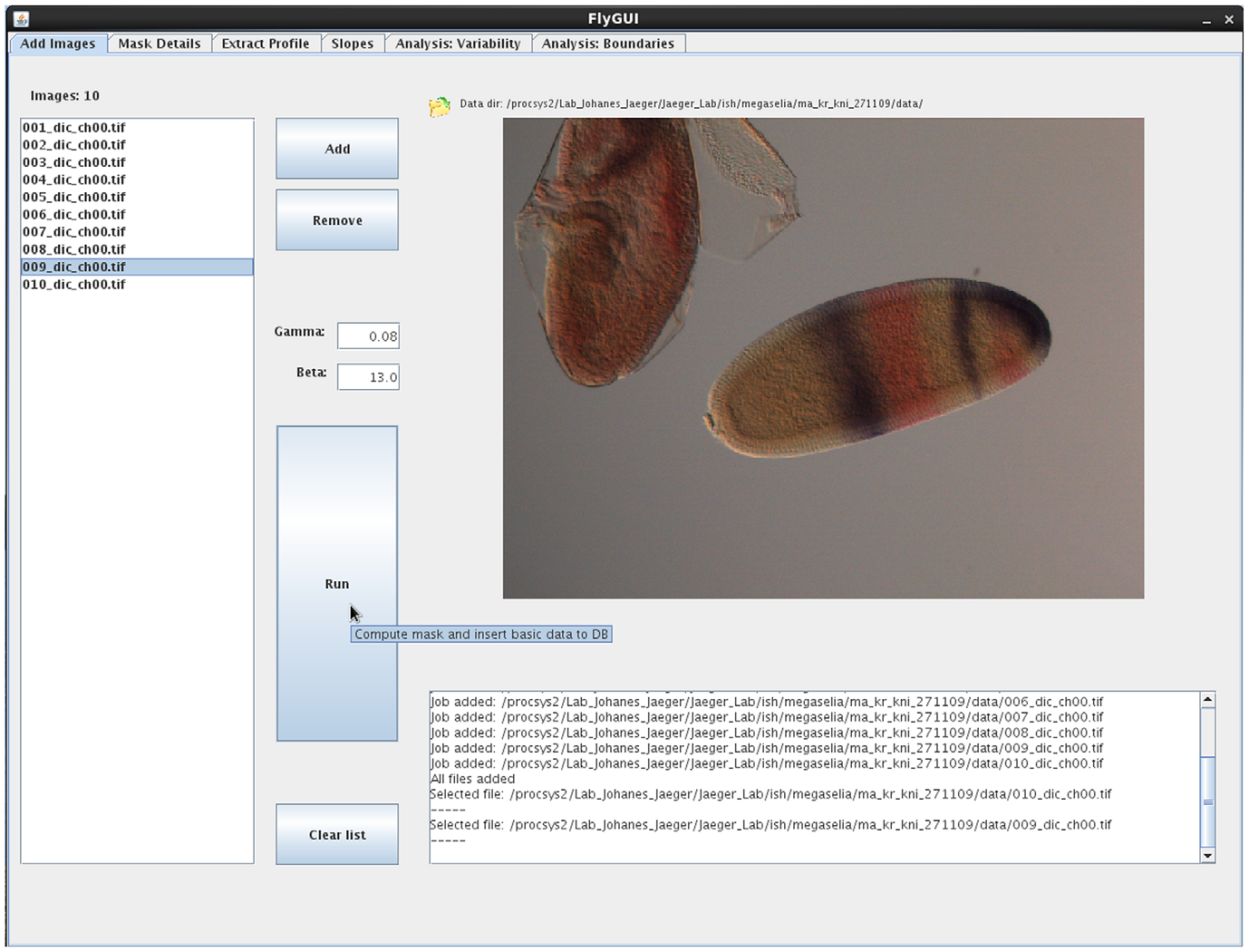
FlyGUI: adding images and creating the embryo mask. Screenshot displaying the ‘Add Images’ tab of our FlyGUI. This tab is used to create embryo masks from selected DIC images. See text for details.

**Figure 6:**
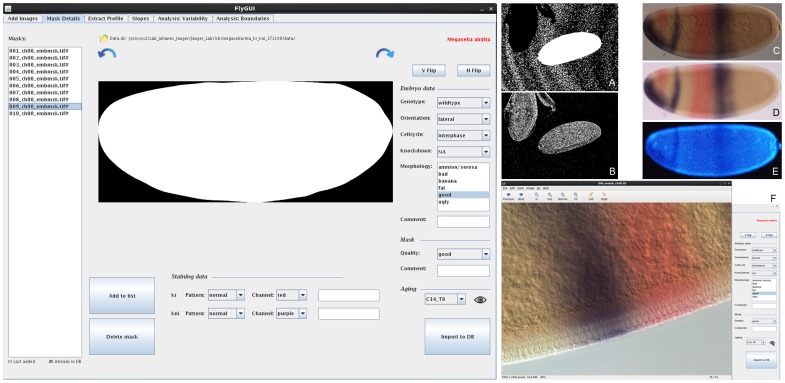
FlyGUI: editing mask details. Screenshot displaying the ‘Mask Details’ tab of our FlyGUI. This tab is used for embryo re-orientation, staging, and quality control for the segmentation process. Different embryo images are displayed by clicking on the curved direction arrows (shown in A–E). (A,B) Intermediate images generated after the ‘edge detection’ and ‘fill holes’ operations in the processing pipeline. (C–E) Cropped and rotated DIC, bright-field, and nuclear counterstain images. (F) Pop-out high-resolution image of membrane morphology. See text for details.

**Figure 7:**
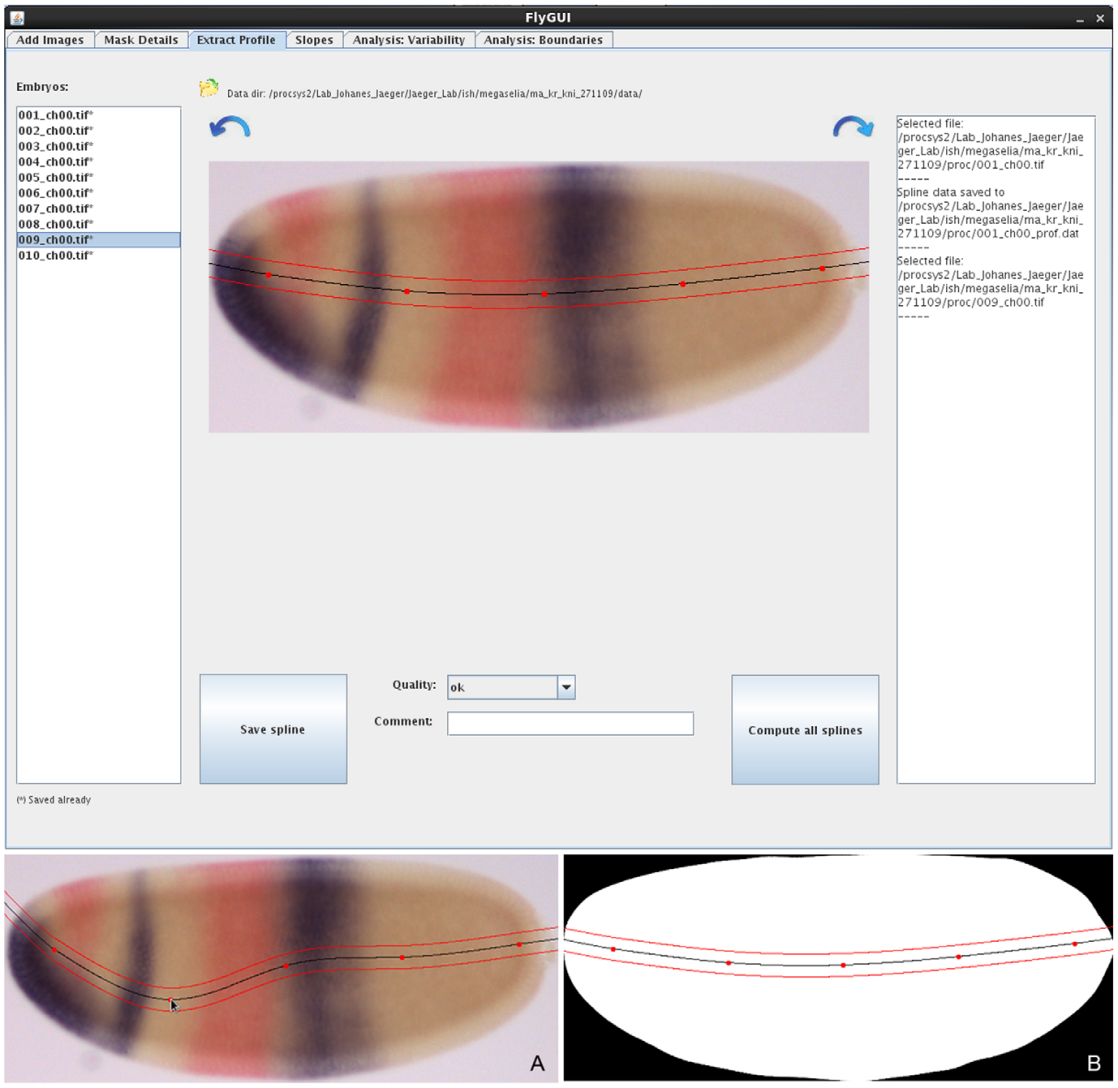
FlyGUI: extracting expression profiles along the A–P axis. Screenshot displaying the ‘Extract Profile’ tab of our FlyGUI. This tab is used to extract expression profiles along the A–P axis. Clicking on the directional arrows switches between bright-field and embryo mask views (A,B). The automatically generated extraction strip can be re-positioned if necessary by clicking and dragging one of the 5 knots of the spline (A). See text for details.

**Figure 8:**
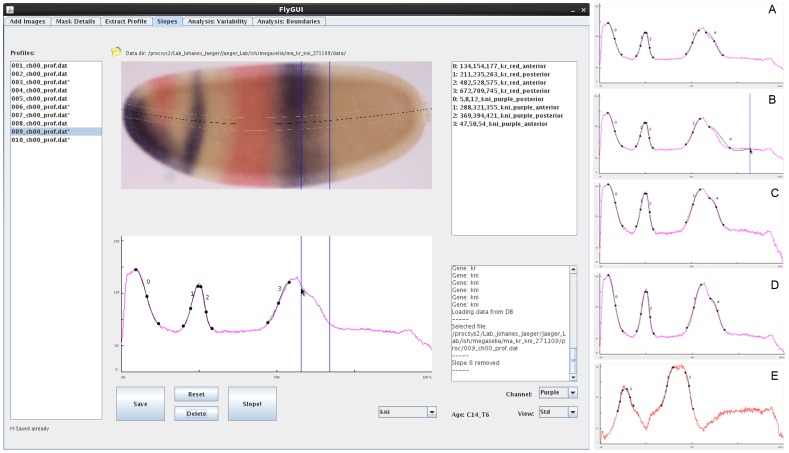
FlyGUI: determining boundary (slope) positions. Screenshot displaying the ‘Slopes’ tab of our FlyGUI. This tab is used to manually identify the boundaries (slopes) present in extracted expression profiles. In the main screen, the boundaries of *kni* slope 4 are being positioned. Once satisfactory spline approximations have been found (A), slopes are added to the database by pressing the ‘Slope!’ button. Boundaries may be re-positioned by clicking and dragging on the end knots of the splines (B). Two additional views of the expression profiles aid separation of signal from background by plotting the logarithm (C), or minimum-to-maximum range (D) of the expression profile. Selecting the ‘Channel’ drop-box allows us to switch expression profile view from purple to red stains (E). See text for details.

**Figure 9:**
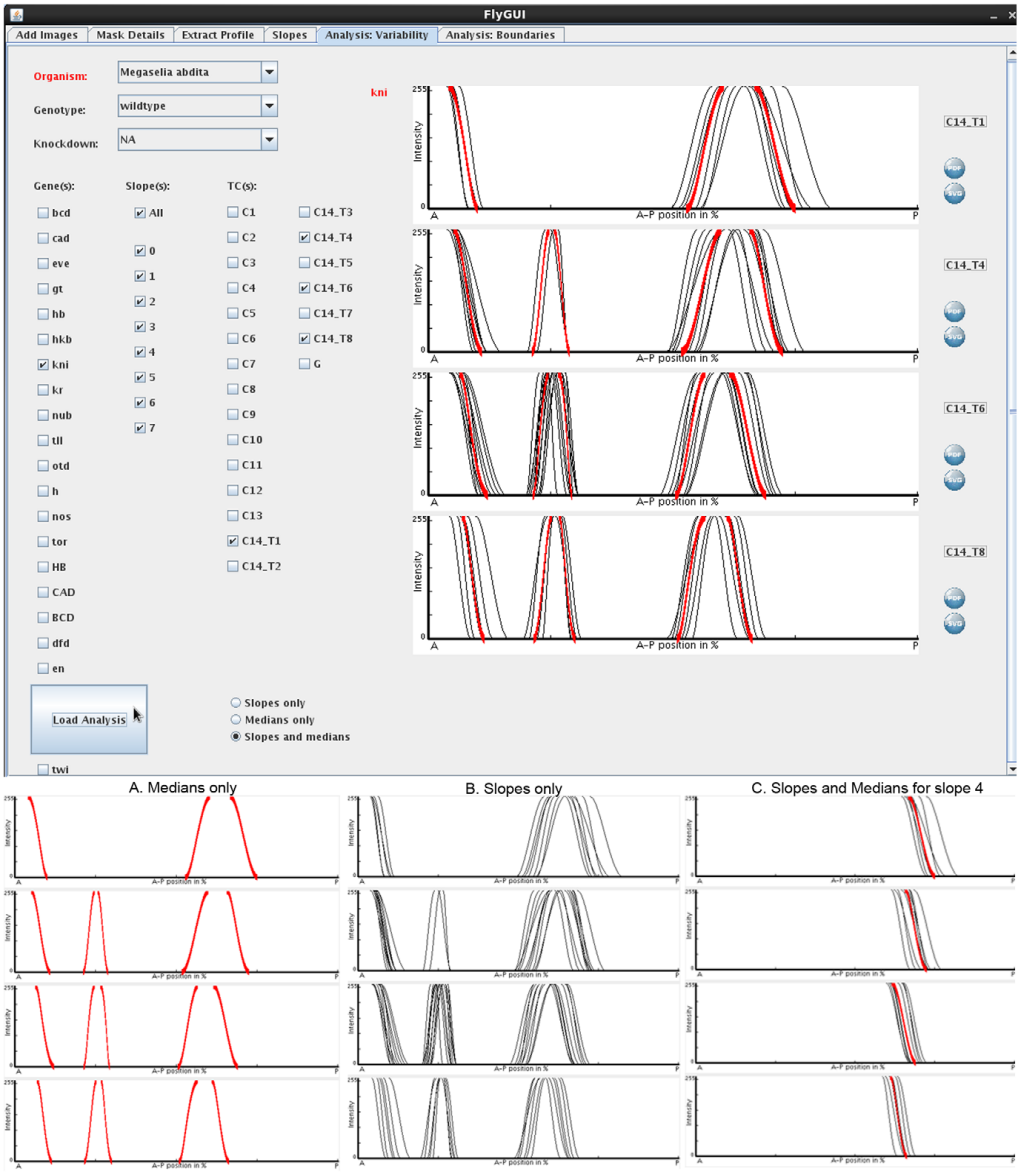
FlyGUI: analysing positional variability. Screenshot displaying the ‘Analysis: Variability’ tab of our FlyGUI. This tab allows us to plot sets of expression boundaries for specific genes and time classes. Individual slopes and medians can be displayed together (main panel), or separately as median-only (A), or as slopes-only (B) graphs. Either entire gene expression patterns (main panel), or individual slopes (C) can be plotted. Median slopes for multiple genes can be combined (not shown). See text for details.

**Figure 10:**
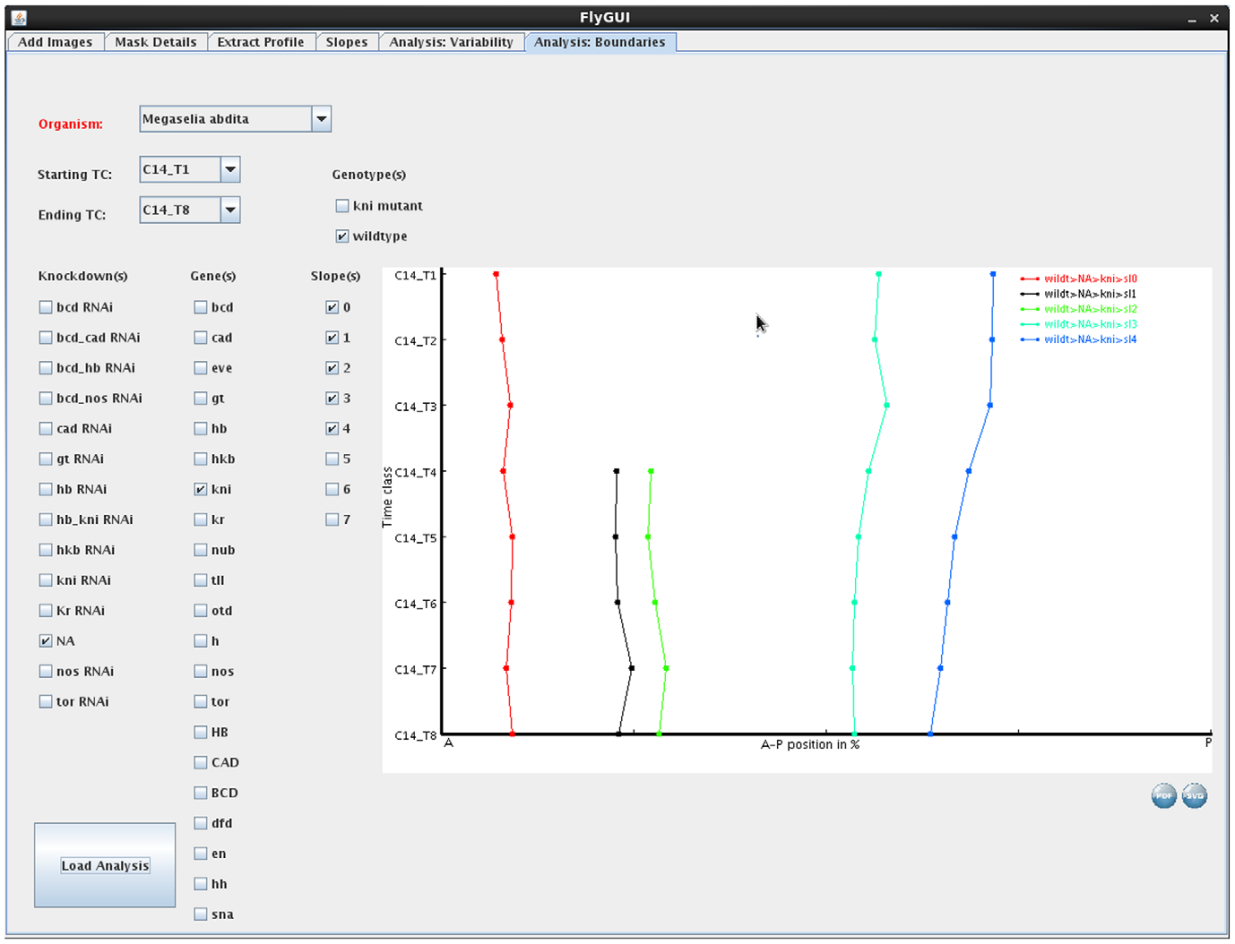
FlyGUI: analysing spatio-temporal dynamics of gene expression. Screenshot displaying the ‘Analysis: Boundaries’ tab of our FlyGUI. This tab allows us to plot median boundary positions through time to visualise spatio-temporal dynamics of gene expression. See text for details.

In this section, we describe how to use FlyGUI to process a batch of stained fly embryos. There are four processing steps in the work flow: (1) adding images, (2) creating the embryo mask, (3) extracting expression profiles, and (4) extracting slopes to identify boundaries. Each of these steps correspond to a separate tab of the FlyGUI (Figs. 5,6,7,8). In addition, there are two graphical analysis methods looking at the variability and dynamics of expression boundaries, each with their own tabs (Figs. 9 and 10).

### Adding Images

In our example we process images from 10 embryos of *M. abdita* stained enzymatically for *kni* transcripts (NBT/BCIP, in purple) and *Kr* transcripts (FastRed, in red; see Fig. 5). Raw images are stored in a folder named ‘data’, while a second folder named ‘proc’ will be the destination for stored processing steps. In our example, each embryos has 4 images: a bright-field, a DIC, a nuclear stain and a high magnification image of the mid-dorsal membrane morphology. These images are labeled, for the first embryo, as ‘001’, ‘001_dic’, ‘001_nuc’ and ‘001_memb’ respectively, and as ‘002’, ‘003’ etc for subsequent embryos. Note that processing is also possible (but slightly more failure-prone) using bright-field or DIC images alone, in case the other images are not available.

In the ‘Add Images’ tab (Fig. 5) clicking the ‘Add’ button opens a file browser dialog window allowing navigation to the data folder. On entering the data folder only the DIC image file names are displayed. Clicking on these file names adds them to the list (Fig. 5, left-hand side). Images can be removed individually from the list by selecting them and clicking the ‘Remove’ button or collectively by clicking the ‘Clear List’ button.

### Creating the Embryo Mask

Clicking the ‘Run’ button starts the process of computing the embryo mask, and inserts basic data on the mask generation process (gamma and beta values, rotation angle, crop information) into the database using default settings. In an output window at the bottom of the screen, messages are displayed that report the progress of the mask computation (Fig. 5). Individual image processing steps for producing the embryo mask from a DIC image, and to crop and rotate the bright-field, DIC, and nuclear images are shown in Fig. 2 and described in detail in Materials and Methods. Once the process is complete, embryo masks can be viewed in the ‘Mask Details’ tab (Fig. 6, left panel). If the mask-making process fails, or masks of low quality are produced, ‘Gamma’ and ‘Beta’ settings can be adjusted. The ‘Gamma’ parameter controls contrast adjustment through gamma correction. ‘Beta’ is our cleaning factor. On the one hand, decreasing this value causes bigger supernumerary blobs to be removed from the mask image that are likely to be processing artifacts (see Materials and Methods for details). On the other hand, if embryos are smaller than typical for the default settings (∼500 µM; this applies, for example, to embryos of *C. albipunctata*), the value of Beta must be increased to prevent the embryo from being considered an artifact and therefore from being removed.

Before embryos are imported into the database they must be correctly orientated and annotated. In the ‘Mask Details’ tab (Fig. 6, left panel), a list of embryos with computed masks is shown on the left-hand side. A mask is displayed in the central panel by clicking on an embryo from the list. Clicking the directional arrows above the mask image will display intermediate images generated after the ‘edge detection’ and ‘fill holes’ operations during the mask-generation process (see Materials and Methods), as well as cropped DIC, nuclear, and bright-field images before cycling back to the embryo mask (Fig.6A–E). This allows us to pinpoint potential problems with mask generation, and enables visual quality control by the user.

The embryo mask, DIC, nuclear, and bright-field images can be re-orientated if necessary by flipping them vertically and horizontally using the ‘V Flip’ and ‘H Flip’ buttons. This manual flipping step is necessary since automated orientation of embryos in A–P and D–V directions is unreliable (see also [3]). Selected masks can be deleted from the list using the “Delete Mask” button. The ‘Add to List’ button adds the embryo back to the import list, in case that we are not completely satisfied with the mask, and we want to repeat the mask-generation process for that embryo with different gamma and/or beta parameters.

Next, embryos and masks need to be annotated. Additional information on each embryo is organised under 4 headings: ‘Embryo Data’, ‘Mask’, ‘Aging’, and ‘Staining Data’. We discuss each in turn below.

‘Embryo Data’ are added using the drop-down menus on the right of the screen (Fig. 6, left panel). A genotype is selected and applied to the entire batch. In this case ‘wild-type’ is chosen. Other annotations use controlled vocabularies (implemented by drop-down menus), and include the orientation of the embryo in the image (‘lateral’, ‘nearly lateral’, and ‘not lateral’), the phase of the cell cycle (‘interphase’, ‘mitosis’, ‘unknown’), knock-down (if the embryo has been subject to RNA interference), and the morphology (‘amnion/serosa present’, ‘bad’, ‘banana’, ‘fat’, ‘good’, ‘ugly’). A comment box is also present in case further details need to be documented.

‘Mask’ quality is assessed by eye and the fit rated as either ‘good’, ‘ok’, or ‘not good’ using the drop-down menu. Again a comment box is present to record any observations that may not fit the controlled vocabulary.

‘Aging’: embryo age is assessed based on the number of nuclei visible in the nuclear counterstain (if available, see Fig. 6E), and membrane morphology (Fig. 6F; based on previous staging schemes established in *D. melanogaster* [3]). Images of detailed membrane morphology are accessed by clicking the eye icon in the ‘Aging’ subheading. This executes the external viewer Eye Of Gnome (http://projects.gnome.org/eog) to display the high-resolution membrane image. Once the time class has been determined, it can be selected from the drop-down menu.

In the ‘Staining Data’ subheading we classify the staining intensity as ‘normal’, ‘weak’, and ‘saturated’. At this step, the colour of the stain for each gene is also selected, and any additional comments on staining quality are entered into the comment boxes.

When completed, annotations are added to the database by clicking the ‘Import to DB’ button.

To refine the staging process we use an additional program (called ‘FlyAGE’) to view embryos stained for particular genes at specific time classes. FlyAGE is used after annotation of the whole batch of embryos is complete (see Fig. 4 and Material and Methods for details of staging). The program displays montages of all bright-field, membrane morphology, or nuclear images from a single stage. This allows the user to compare embryos between and within time classes, and to spot outliers or mis-classified embryos. The latter can be reassigned to other time classes without having to return to FlyGUI. This visual staging process is always implemented by at least two independent experts to avoid biases in classification.

### Profile Extraction

In the ‘Extract Profile’ tab (Fig. 7, top panel) we select a stripe of 10% D–V width along the A–P axis of the embryo, and extract expression data from that stripe. Clicking on an embryo retrieves profile data from the database (if any). If no data is present for that embryo, a new spline is calculated in A–P direction following the midline of the embryo (see Fig. 3, and Materials and Methods for details). Information on this process is displayed in an output panel at the right-hand side of the window (Fig. 7, top panel). The resulting extraction stripe is displayed as an overlay on the bright-field image. Clicking the directional arrows alternates between overlays using bright-field and embryo mask images (Fig 7. A, B). Five equidistant points (or knots) are placed along the profile. These knots can be dragged using the mouse to manually reposition the extraction stripe if necessary (Fig. 7A). The resulting spline curve is saved to the database by clicking the ‘Save spline’ button. It is used to calculate expression profiles under the area of the extraction stripe (see Materials and Methods). Alternatively, profiles can be computed and saved automatically for the entire batch of embryos in an unsupervised mode by clicking ‘Compute all splines’. If a good spline cannot be created, the quality can be changed from ‘good’ to ‘ok’ or ‘not good’ in the drop-down menu. Again, one can also add comments.

### Extracting Slopes

The ‘Slopes’ tab of FlyGUI (Fig. 8, left panel) implements the manual fitting of spline curves to the expression profiles calculated in the previous step. These splines are used to identify the gene expression domain boundaries (‘slopes’) we observe in a profile. A list of profiles is displayed on the left of the screen. Clicking on a profile displays the associated bright-field embryo image with its overlayed extraction stripe from the previous processing step. Below the embryo, the extracted expression profile is shown. Processing information is displayed in an output window at the bottom right of the screen (Fig. 8, left panel). To fit splines to the profile, the channel (red or purple) is selected from a pull-down menu, followed by the gene. In our example we start with *kni* in the purple channel.

Boundaries (slopes) are added by clicking once on the peak of the expression profile, and once on the position where the levels of expression can no longer be detected. Each left mouse click positions a blue bar overlapping the expression profile and the bright-field image (Fig. 8, left panel). The bar can be removed by clicking the right mouse button, and repositioned by clicking the left again. When the two bars are positioned correctly, clicking the ‘Slope!’ button draws a spline between the points (Fig. 8A). Additional adjustments can then be made to the spline by dragging the start or end knot (Fig. 8B). Three different views of the expression profile are available: the standard view (Fig. 8, left panel), a log-intensity plot (Fig. 8C), and a minimum-to-maximum-intensity plot (which is equivalent to the standard view, scaled to maximum and minimum values of the profile; Fig. 8D). Depending on the quality of staining, the different views help to emphasize the signal in order to better distinguish staining from background.

Typically, multiple splines are fit to one profile, one for each expression boundary. Each spline is displayed in the top right-hand panel, and can be removed again by highlighting, and clicking the ‘Delete’ button (Fig. 8, left panel). Additionally, a spline can be assigned a higher or lower identification number by clicking the right or left mouse button, while holding the Ctrl (control) key. Switching the channel to ‘red’ allows the extraction of boundaries from a second stain, in our case *Kr* (Fig. 8E). In the end, if necessary, all splines can be removed by clicking the ‘Reset’ button. Otherwise, information on each spline individually is saved in the database.

The identification of gene expression boundaries is the only explicitly manual step in our workflow. This implies that user performance has an influence on measured boundary positions. We minimize user-introduced error by (1) the use of multiple embryos per time point to establish the median position of an expression domain boundary, and (2) a set of guidelines for the user for determining boundary positions.

These guidelines are as follows: a gene expression domain boundary has an upper and lower limit of expression intensity (expression level), where the upper one is placed where the gene expression signal levels off, and the lower limit is placed where signal is no longer distinguishable from background. Finally, we ensure that the positions we select in the profile graph agree well with the expression boundaries visible in the bright-field image.

### Analysis: Variability

The ‘Analysis: Variability’ tab in FlyGUI (Fig. 9) allows us to view the splines added in the previous step, and to assess their embryo-to-embryo variability and positioning with respect to each other. ‘Organism’, ‘genotype’, and ‘knock-down’ can be selected from the drop-down menus on the top left of the screen. In our example, we then select ‘*kni*’ as the gene, ‘all’ for the slopes, and time classes ‘C14_T1, T4, T6 and T8’ marking the corresponding tick boxes on the right (Fig. 9). Splines are viewed as ‘slopes and medians’ (Fig. 9, top panel), or as ‘medians only’ (Fig. 9A), or ‘slopes only’ (Fig. 9B) by selecting the right radio button at the bottom of the screen, and clicking the ‘Load Analysis’ button. Medians are displayed as a solid line, coloured differently for each gene (red in the case of *kni*, Fig. 9). Only median splines can be displayed if multiple genes are viewed simultaneously. Individual plots can be exported in SVG or PDF format by clicking the corresponding icons on the right of the plots.

### Analysis: Boundaries

The ‘Analysis: Slopes’ tab of FlyGUI (Fig. 10) allows the boundary position of individual slopes to be plotted across time and space. ‘Organism’ and the range of time points to be plotted can be selected from the drop-down menus at the top of the screen. Checkboxes allow ‘knock-downs’, ‘genes’, and ‘slopes’ to be selected. Clicking the ‘Load Analysis’ button plots boundary positions. In the example shown, we have selected ‘*Megaselia abdita*’ as the species, time classes ‘C14_T1 to T14_T8’, a wild-type genotype with no knock-down (‘NA’), displaying all slopes for the gene ‘*kni*’ (Fig. 10). Again the plot can be exported in SVG or PDF format by clicking one of the icons below the plot.

### Future Improvements

The current version of FlyGUI has allowed us to process a large number of embryo images (approximately 3′300 so far) from four different species of dipterans. It is designed for simplicity and ease of use. Although the workflow is largely automated, it still requires a few manual processing steps, such as flipping embryo orientation, time classification, and boundary extraction using splines. This is because automatic orientation of embryo masks is notoriously difficult due to significant embryo shape variation, and a lack of pronounced asymmetry along both A–P and D–V axes, while the high and uneven background in bright-field or DIC images makes automatic recognition of boundaries non-trivial. To resolve these issues remains a major challenge for future work.

Another challenge is the measurement of relative expression levels from enzymatically stained embryos. We currently only measure boundary positions but not relative expression levels of different domains. One reason for this is that our current protocol involves potentially non-linear amplification of the signal, and it is difficult to detect and avoid saturation in the opaque precipitate of the NBT/BCIP stain. Another reason is the large variability in background staining and illumination in the images we use, which makes it difficult to separate signal from noise. Further research will be required to solve these problems.

Furthermore, we are exploring possibilities of processing data from more species (dipteran and non-dipteran), and data sets created by other research groups. This raises the challenge of creating embryo masks from non-standardised data meaning that our method should be made more robust when dealing with lower quality images (e.g. high and variable background, images crowded with multiple embryos etc.), and embryos imaged in different manners (e.g. greyscale images from publications, or DIC/bright-field images only). This should be achievable by using alternative segmentation algorithms such as those based on machine learning strategies proposed in [29], which is based on a statistical model of embryo shape, and its principal components of variation. It would be straightforward to combine such an algorithm with our profile and boundary extraction methods.

Finally, our method can be easily adapted to any system in which graded gene expression patterns need to be measured along a well-defined axis. Examples of such systems are the D–V system and wing imaginal disk of *Drosophila* (refs). With appropriate modifications to image segmentation and axis identification algorithms, our profile and boundary extraction methods could also be applied in these contexts.

## Conclusions

In this paper, we have presented a robust, “medium-throughput” method for measuring the position of graded gene expression domain boundaries along the A–P axis of different dipteran embryos. Our method fills a gap between previously published methods that either provide very precise, high-resolution measurements of expression levels across time and space, or enable the high-throughput extraction, analysis, and comparison of expression patterns from thousands of embryo images in genome-wide expression databases. We have used this method to measure the spatio-temporal dynamics of maternal and gap gene expression in four different dipteran species. We have shown elsewhere that the resulting integrated data sets can be used to analyse and reverse-engineer the structure and dynamics of the gap gene network [31]. We are now using our processing pipeline to process and analyse data from mutants and gene knock-downs by RNA interference (RNAi). Our method is easily adaptable to other developmental contexts and species, which require the measurement of domain boundary positions along a well-defined axis. This makes it widely applicable for network-level analyses of developmental regulatory systems based on quantitative spatio-temporal gene expression data.
